# Eight Days of L-Citrulline or L-Arginine Supplementation Did Not Improve 200-m and 100-m Swimming Time Trials

**DOI:** 10.3390/ijerph19084462

**Published:** 2022-04-07

**Authors:** Ozcan Esen, Mustafa Can Eser, Mekki Abdioglu, Daniela Benesova, Tomasz Gabrys, Raci Karayigit

**Affiliations:** 1 Department of Health Professions, Manchester Metropolitan University, Manchester M15 6GX, UK; 2 Institute of Sport, Manchester Metropolitan University, Manchester M155TN, UK; 3 Faculty of Sport Sciences, Ankara University, Gölbaşı, Ankara 06830, Turkey; mceser@ankara.edu.tr (M.C.E.); mekkiabdioglu@gmail.com (M.A.); 4 Sport Centrum Faculty of Pedagogy, University of West Bohemia, 30100 Pilsen, Czech Republic; dbenesov@ktv.zcu.cz (D.B.); tomaszek1960@o2.pl (T.G.)

**Keywords:** nitric oxide, ergogenic aid, sport nutrition, supplements, functional foods

## Abstract

The effects of L-citrulline or L-arginine supplementation on exercise performance are equivocal, and the effects on swimming performance are unclear. We aimed to assess whether 8-day supplementation with L-arginine or L-citrulline supplementation would improve 200 m and 100 m freestyle swimming time-trial performances. After the baseline trial (first visit), in a double-blind, randomised design, 15 trained/developmental (5 females) swimmers and triathletes were assigned to three groups and underwent an 8-day supplementation period, with a daily dose of either 8 gr L-arginine (Arg, *n* = 5) or L-citrulline (Cit, *n* = 5) or placebo (Pla, *n* = 5). On day 9, participants completed experimental trial (second visit). In each trial, after blood sampling, participants performed both 200 m and 100 m freestyle swimming time-trials, with 30 min recovery between trials. Plasma nitric oxide (NOx) and blood lactate concentrations (BLa) were collected immediately before and after 200 m and 100 m TTs, respectively. No significant difference was observed in NOx between groups (*p* = 0.201). There was no significant difference in 200 m (*p* = 0.226) and 100 m swimming time-trials (*p* = 0.993) between groups. There was a main effect of time on BLa concentration (*p* < 0.001), but no trial × group (*p* = 0.243) and trial × lactate × group interaction effect (*p* = 0.276) was present. Furthermore, 8-day either L-citrulline or L-arginine supplementation did not enhance middle (200 m) and short-distance (100 m) swimming performance in trained/developmental swimmers and triathletes. These findings do not support the use of L-citrulline or L-arginine supplementation as ergogenic aids for swimming performance.

## 1. Introduction

Nitric oxide (NO) is an important physiological signalling molecule for skeletal muscle perfusion, metabolism, contractility, and fatigue resistance [1,2], and it is attracting much interest in sport physiology and nutrition as an ergogenic aid [3]. To date, many studies have shown that dietary NO related supplements, such as nitrate-rich beetroot juice or watermelon juice, enhance athletic performance [4,5,6]. NO has been suggested to improve exercise performance primarily by enhancing exercise induced vasodilation and increasing the oxygenation status in the working muscles [7].

L-Citrulline (L-Cit) and L-Arginine (L-Arg) are two amino acids that have been shown to improve athletic performance (i.e., greater power output and time to exhaustion) mainly based on increased NO production [8]. L-Arg content is high in seafood, watermelon juice, nuts, seeds, algae, rice protein, meats, and soy protein [8]. L-Cit is primarily found in cucumbers, watermelon and other melons [8]. Additionally, L-Cit is also produced endogenously by two main pathways: firstly, synthesized from glutamine that produces ornithine in enterocytes and then by concentration of ornithine and carbamoylphosphate in a reaction catalysed by ornithine carbamoyl transferase [8] and secondly, produced by the conversion of L-Arg to NO in a reaction catalysed by NO synthase (NOS) enzymes [9]. In human metabolism, NO synthesis mostly occurs through oxidation of L-Arg by NOS enzymes to L-Cit (NOS-dependent) [10], but it can also be produced by dietary nitrate intake via the reduction of nitrate to nitrite (NOS-independent) [1]. While the literature has mostly focused on nitrate supplementation in the last decade [11], numerous NO-stimulating supplements in the sport nutrition market containing L-Arg claim performance and health benefits despite unclear and contrary findings of the effects of L-Arg supplementation on performance and health variables [12,13].

Whilst improved exercise capacity was attributed to the elevated NO level following L-Arg supplementation in a previous study [12], Vanhatalo et al. [13] reported no effect in NO level (a sensitive marker of NO) or exercise capacity. Together, these findings suggest that a potential ergogenic effect of L-Arg likely depends on whether it elevates plasma NO level. Given that orally ingested L-Arg goes under significant pre-systematic and systematic breakdown [14,15], this limits L-Arg availability in the systematic circulation [16]. L-Cit is an end product of NOS activity during NO synthesis and was shown to recycle into L-Arg in a subsequent of NO production [17,18]. Additionally, L-Cit supplementation was reported to elevate systematic and muscle L-Arg level higher than L-Arg supplementation [19] and therefore provide higher NOS activity and NO biomarkers in plasma compared to L-Arg supplementation [19,20,21]. Consequently, L-Cit might be a better ergogenic than L-Arg for physical performance [22]. There is also evidence to suggest that short-term L-Cit supplementation may enhance skeletal muscle oxygenation [20], exercise performance [20,23,24,25,26,27,28], and fatigue resistance [20]. Moreover, Stanelle et al. [29] reported that L-Cit supplementation might cause a slight enhancement in the cycling performance of well-trained athletes. It was also shown that, due to exercise induced muscle damage, L-Cit supplementation can decrease serum creatine kinase concentrations along with increasing recovery [3]. According to some studies, L-Cit supplementation reduces blood lactate (BLa) concentrations and muscle pain 24 h post-exercise [29,30], which might be related to its antioxidant features. Hence, L-Cit supplementation might be an alternative dietary intervention to elevate NO production and therefore to enhance exercise performance.

High-intensity exercise causes an accumulation of ammonia in the blood and results in the phosphofructokinase activation, which improves the rate of glycolysis [31]. BLa increases as the glycolysis rate is increased during high-intensity exercise and causes fatigue [31]. By buffering ammonia through the urea cycle, L-Cit supplementation is expected to enhance the aerobic utilization of pyruvate, thus decreasing lactate production via the anaerobic pathway [24]. Therefore, we can expect that L-Cit and L-Arg supplementation would improve high-intensity (e.g., 100 m and 200 m) swimming performance.

Those previous L-Cit and L- Arg related studies have been applied to cycling [25,32,33,34,35], walking [23], and running [20,24] whereas only one study has investigated the effects of L-Cit supplementation on swimming performance, reporting a faster swimming time in a high-intensity interval protocol in young swimmers [36]. However, since L-Cit was combined with various other compounds (L-Arg and branch chain amino acids [BCAA]) and a tolerance related exercise protocol was used in that study by Hsueh et al., it is difficult to draw a solid conclusion regarding whether improved swimming performance following L-Cit supplementation is related to elevated NO production [36]. Taken together, it is still unclear whether L-Cit or L-Arg supplementation would improve swimming performance by increasing NO production via the NOS-dependent pathway.

Therefore, the aim of this study was to assess whether 8-day supplementation with L-Arg or L-Cit supplementation would improve 200 m and 100 m freestyle swimming time-trial performances.

## 2. Materials and Methods

### 2.1. Participants

Fifteen, trained/developmental [37], young adult male (10) and female (5) swimmers and triathletes (mean ± SD: age 25 ± 7 years, height 177.0 ± 6.3 cm, body mass 78.0 ± 12.3 kg) participated in this study. Participants were assigned to three groups (5 participants per group), and each group consisted of 3 swimmers and 2 triathletes in order to provide an even distribution in terms of the fitness levels of the groups. An a priori sample size calculation was conducted by using G*Power software (Version 3.1) based on the effect size (0.69) of a previously published study investigating the impact of L-Cit supplementation on exercise tolerance during severe-intensity cycling exercise [20]. F-test family was used with repeated measures, within-between interaction, α = 0.05 and a power = 0.80 indicated that 9 participants would be required. All participants had at least 5 years’ experience competing in regional and university-level competitions and completed at least 3-day weekly swimming training sessions (swimmers: 6–8 h; triathletes: 4–6 h a week). All female participants in this study were using hormonal contraceptives. Participants provided their dietary record 24 h before the first trial and repeated the same diet 24 h before the subsequent trial. Participants were also asked to refrain from high-intensity exercise and consumption of alcohol, caffeine, nutritional supplements, and anti-inflammatory drugs 24 h before each trial. All participants provided written informed consent and health screening forms before participating in the study. Ethical approval was received from the Faculty of Medicine, Dentistry and Clinical Sciences Research Ethics Committee at the University of Chester (reference no: 1191/16/OZ/CSN).

### 2.2. Experimental Design and Supplementation

Participants were required to visit to the laboratory and swimming pool on two separate occasions 9 days apart. On each visit, following venous blood sample collection, participants completed a 200 m front-crawl TT. Then, participants performed 100 m front-crawl TT followed by 30 min of passive recovery. After completion of first (baseline) trial, participants were assigned into three groups for supplementation of either L-arginine, (Arg, *n* = 5), L-citrulline (Cit, *n* = 5), or placebo (Pla, *n* = 5) for 8 days, in a randomized, double-blind design.

An independent technician, not otherwise involved in this study, prepared the supplements. The supplements were provided in a small colourless bag containing 8 g of powder and were consumed every morning (8–10 am) over the first 7 days. On the final day of supplementation, 8 g of powder was ingested 1.5 h before the 200 m TT. Pure cellulose, arginine, and citrulline powders (same colour and tasteless) (NOW Sports Nutrition, NOW Foods, Bloomingdale, IL, USA) were consumed by mixing with 500 mL of water. Participants were instructed to set up a time reminder for their daily supplement intake and asked to keep a record of any days when they missed taking the supplement in order to monitor supplement compliance. Participants were reminded their supplement intake time daily via email.

Upon arrival at the laboratory and following 10 min of rest, a venous blood sample (~6 mL) was collected into red top serum tube. Samples were then centrifuged at 1160 g and 4 °C for 10 min (Hettich^®^ 320 centrifuge, Montreal, QC, Canada). Plasma was subsequently aliquoted and stored in labelled tubes at −80 °C until analysis. Before analysis, samples were deproteinised to minimise assay interference. This was done by using ultrafiltration with 10,000 MW cut of filters (Sartorius™ Vivaspin™ 500 Centrifugal Concentrators, USA). Filters were washed with deionised water before use. Samples were then spun at 10,000 g for 10 min. The run through was then collected for analysis directly. All samples were analysed with a colorimetric assay kit (Total Nitric Oxide and Nitrate/Nitrite Colorimetric Assay Kit, Parameter^TM^, R&D Systems KGE001, Minneapolis, MI, USA) using the Griess reaction (Green et al., 1982). Briefly, plasma NOx was measured; to 50 µL of sample, 25 µL of NADH and 25 µL of nitrate reductase was added, and the mix was incubated in the well plates for 30 min at 37 °C. A volume of 50 µL of Griess Reagent I and Greiss reagent II was added to each well and incubated for a further 10 min at room temperature, and then, the optical density was determined at 562 nm using the EZ read 400-microplate reader.

Capillary BLa was also measured using a lactate analyser (Lactatepro©, Arkay, Kyoto, Japan) from finger pinprick samples. BLa was measured immediately before and after the 200 m and 100 m freestyle swimming performance trials.

### 2.3. Simulated Swimming Time Trials

All trials took place in the same indoor swimming pool (1–3 m dept, 25 m length, 12.5 m width, and 28 °C water temperature), with trials completed at the same time of day of each trial (10–12 pm). The swimming TTs protocol adopted from Lindh et al. [38] was used to create a situation as close as real swimming competition and consisted of 200 m and 100 m front-crawl swimming distances. A standardized low-to-moderate intensity warm-up (20–25 min) was applied prior to each trial. Ten min following to warm-up, participants completed a 200 m freestyle TT. Then, the participants recovered in a seated position for 30 min and were only allowed to drink water, which was recorded and precisely replicated on the 2nd trial. After 30 min recovery, 100 m TT was performed. All TTs were began with diving start from diving box and timed with a stopwatch.

### 2.4. Statistical Analysis

Commercially available software SPSS 27.0 (IBM Corp., Armonk, NY, USA) was used for statistical analysis. Two-way (Mixed model) ANOVA was used to assess between-supplement differences in plasma NOx level and TT performances. The three-way ANOVA (trial × lactate × group) was also applied to assess between-supplement differences blood lactate concentration. Sphericity was analysed by Mauchly’s test of sphericity followed by the Greenhouse–Geisser adjustment where required. If any differences were detected, post hoc Bonferroni adjustment was applied. The level of significance was accepted as *p* < 0.05, and data are presented as mean ± SD.

## 3. Results

**Figure 1:**
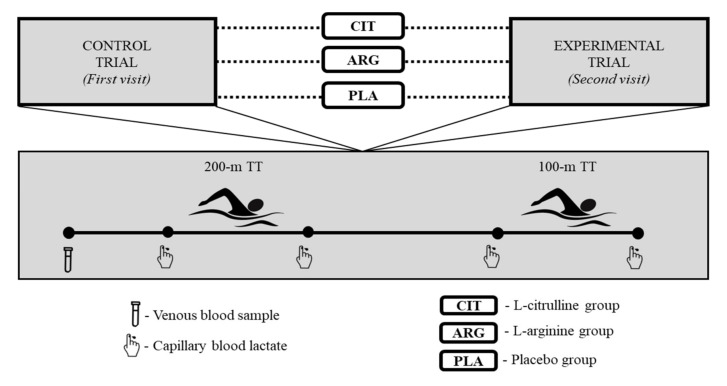
Experimental design.

**Figure 2:**
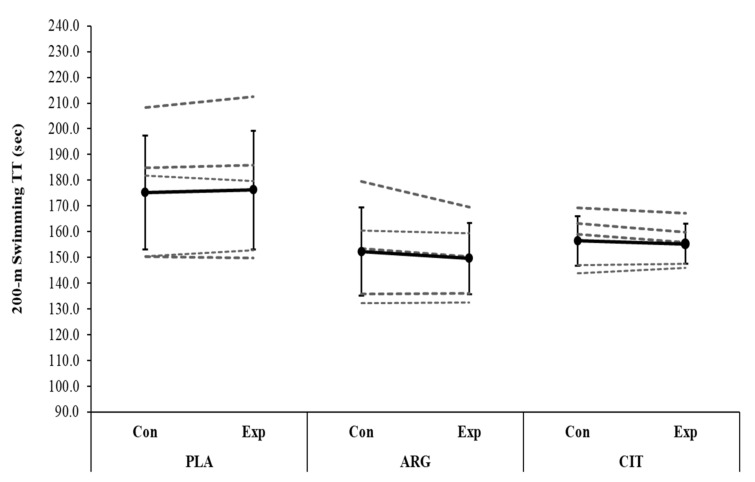
Group mean (SD) and individual 200 m swimming TT responses after 8-day L-citrulline or L-arginine or placebo supplementation are shown in the black and dashed lines, respectively. PLA, placebo; ARG, L-arginine; CIT, L-citrulline; Con, control trial; Exp, experimental trial; TT, time trial.

**Figure 3:**
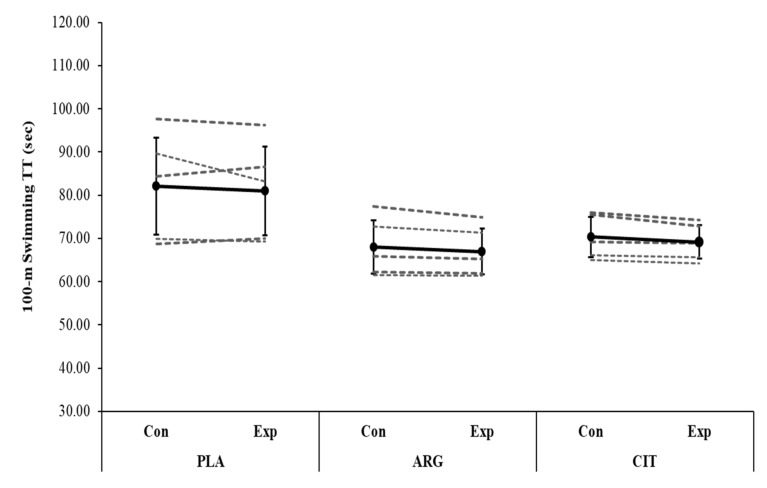
Group mean (SD) and individual 100 m swimming TT responses after 8-day L-citrulline or L-arginine or placebo supplementation are shown in the black and dashed lines, respectively. PLA, placebo; ARG, L-arginine; CIT, L-citrulline; Con, control trial; Exp, experimental trial; TT, time trial.

No significance difference was observed in NOx between trials (*F* = 1.49; *p* = 0.155; ŋ_p_^2^ = 0.238) and between groups (*F* = 1.59; *p* = 0.201, ŋ_p_^2^ = 0.212, Table 1). There was no significant difference in 200 m TT between trials (*F* = 1.44; *p* = 0.254; ŋ_p_^2^ = 0.107) and between groups (*F* = 1.69; *p* = 0.226; ŋ_p_^2^ = 0.220, Figure 1). Likewise, there was no significant difference in 100 m TT between trials (*F* = 3.83; *p* = 0.074; ŋ_p_^2^ = 0.242) and between groups (*F* = 0.01; *p* = 0.993; ŋ_p_^2^ = 0.001, Figure 2). There was a main effect of time on BLa concentration (*F* = 155.01; *p* < 0.001; ŋ_p_^2^ = 0.928), but no trial × group (*F* = 1.59; *p* = 0.243; ŋ_p_^2^ = 0.210, Figure 3) and trial × lactate × group interaction effect (*F* = 1.35; *p* = 0.276; ŋ_p_^2^ = 0.184, Table 1) was present.

## 4. Discussion

The aim of this study was to assess the efficacy of nutritional supplementation with L-Cit and L-Arg as an ergogenic aid that would improve high-intensity swimming TT performances. The original findings of the present study are that 8-day of either L-Arg or L-Cit supplementation had no effect in NOx levels, 200 m or 100 m swimming TTs and BLa in trained/developmental athletes compared with placebo. These findings do not support either L-Cit or L-Arg supplementation as an ergogenic aid for trained/developmental swimmers and triathletes over 100 m and 200 m swimming distance.

Both acute [34,39,40,41,42] and chronic supplementation (>7 days) [19,20,26,27,28,43] with L-Cit have been previously reported to improve vasodilation by facilitating NO generation. L-Cit dosages between 2.4g [25] and 12g [44] have been used in studies to date, and both doses have been shown to have positive effects on athletic performance. Therefore, it might be admitted that the L-Cit dosage we used (8 g/day) is enough to boost athletic performance. However, in the present study, neither L-Cit or L-Arg supplementation increased plasma NOx, which is in contrast with a previous study that applied 6 g for each supplement for 7 days on recreationally active men [20]. In another study with 5.6 g × 7 days L-Cit supplementation, the plasma NOx increased significantly compared to the placebo group [27]. The most obvious explanation for the discrepant findings between the present and Bailey et al. study [20] is the difference in NO markers; we measured total NOx as a single marker in the present study, whereas Bailey et al. [20] specifically measured plasma nitrite level. Although total NOx is one of the common assessments that have been used as a marker of NO [24,44,45], plasma NO_2_^−^ has been more recently considered more accurate assessment of NOS-derived NO production [12] despite the fact that it is still an indirect assessment for analysis of NO production. As such, further research should be conducted by measuring plasma NO_2_^−^ as it can better reflect human NOS activity than plasma NOx [12].

Our results showed that swimming TT performances were similar between Cit, Arg, and Pla groups compared to their baselines. Some recent studies reported improved exercise tolerance [20,23] and time-to-exhaustion performance [25] with L-Cit supplementation, whereas some others observed no improvement in time-to-exhaustion [35], aerobic and anaerobic exercise performances [24] or a reduction in incremental exercise performance [44] following L-Cit supplementation. These inter-study differences might be linked to differences in the L-Cit supplementation procedures such as duration (acute vs. short-term), type (powder vs. watermelon juice) or type of exercise (running, swimming etc.) Unaltered swimming performances following 8-day supplementation in the present study contrast with the only previous study by Hsueh et al. [36] that reported enhanced swimming times during 8×50 m high-intensity interval swimming protocol after acute supplementation. The most plausible explanation is that this could be related to the differences in the administration of L-Cit supplementation between the two investigations. Hsueh et al. [36] applied L-Cit supplementation with a combination of L-Arg and BCAA, and therefore, it is possible that improved exercise capacity might be due to these other compounds. Indeed, Hsueh et al. [36] did not measure any NO biomarkers and attributed enhanced swimming performance to increased plasma BCAA concentrations. In the present study, even if it is not statistically significant, we found an upward trend on NOx levels between control and experimental trials of both supplement groups (44.1 ± 19.3 vs. 66.4 ± 6.7 mmol/L^−1^ for ARG; and 42.5 ± 1.2 vs. 58.4 ± 7.3 mmol/L^−1^ for CIT). The absence effect of L-Cit and L-Arg supplementation on swimming TT is likely due to insufficiency of these slight increases in NOx considering the potential effect of those supplementations on exercise performance appears to be linked to their impact on NO bioavailability [12,20]. Suzuki et al. [25] observed that L-Cit supplementation with 2.4 g/day over 7-day produced a significant improvement in 4 km cycling TT and subjective feelings of muscle fatigue and concentration. Considering completion time of the given distance, the physiological demands of the trial (~10 min) assessed by Suzuki et al. [25] would have differed compared to those of 100 m and 200 m swimming trials (~1–3 min, respectively). Therefore, we cannot exclude the possibility that the NOS-dependent pathway via L-Arg or L- Cit supplementation may be ergogenic for events where the distance/duration times is longer than 3 min, even with a lower dose of supplementation. Therefore, further research is required to assess the potential ergogenic effects of L-Cit and L-Arg supplementation in longer duration/distances, such as 800 m and 1500 m (7 and 16 min, respectively).

Our findings showed that there was no difference in BLa before or after TTs between groups, which is consistent with previous studies [20,32,33]. In the study of Terasawa et al. [3], for both the L-Cit and Pla groups, the amount of NOx, on day 7, at the time of post-exercise significantly increased compared to pre-exercise while there was no difference on day 0 [3]. While we observed no change in BLa after high-intensity exercise with L-Cit supplementation in this study, previous animal studies have reported lower post exercise BLa and ammonia concentration [46], as well as a lower rate of muscle PCr reduction [47] with L-Cit. Together, these findings suggest that L-Cit supplementation might increase energy contribution from oxidative metabolism, thereby limiting the usage of limited anaerobic energy reserves and reducing the accumulation of metabolites linked to the process of fatigue. The 100 m and 200 m swimming TTs specifically depend on anaerobic energy reserves [48], and given that L-Cit and L-Arg supplementation may enhance oxidative metabolism and therefore limit the use of the finite anaerobic energy reserves [20], this might partly explain the absence of effects of supplementation on BLa concentrations in the present study.

The type of exercise (i.e., swimming) distinguishes this study from other L-Cit and L-Arg studies. Swimming requires more muscle participation than land-based exercises (i.e., walking or running). A main strength of the present study is the application of a time-trial exercise protocol instead of the time-to-exhaustion (TTE) test. Given that there is no competitive sports event in which competition is based on time and distance before exhaustion, TTE tests have limitations in terms of physiological validity [49]. It has also been reported that no association exists with time-to-exhaustion and actual performance [50]. Thus, we used a TT test by applying a ‘real-world’ competition situation [38]. Another strength of our study is the placebo controlled, randomised, and double-blind design. Nevertheless, some limitations must be addressed. Firstly, we were unable to assess plasma L-Cit and L-Arg concentrations due to the low budget, yet previous studies have shown an increase in plasma L-Cit and L-Arg concentrations with doses higher than 6 g/day [34,51]. Another limitation is the small number of participants for a parallel group design. Although there was a good (*n* =15) and higher that required sample size (*n* = 9, based on G*Power calculation) in the present study, it would be better to conduct a crossover study to eliminate the larger inter-individual fluctuations, and far more statistical power would have been provided if all 15 participants had completed the three interventions compared to 5 subjects completing one of the interventions as the small changes in a parallel group design tend to be masked by the large heterogeneity in one group. Therefore, further research should assess the potential effect of L-Cit or/and L-Arg supplementation in swimming time-trial performance with a crossover design. Additionally, the hand timing method can be considered as a limitation as it might have caused to miss small performance changes in swimming TTs in the present study. All our female participants were actively using hormonal contraceptives, which maintain female sex hormones at relatively constant levels throughout the menstrual cycle [52], which would minimise any effect of natural fluctuations in these hormones on physical performance [53]. However, since we did not compare hormone concentrations within the females between conditions, this can be considered as a limitation of the present study.

## 5. Conclusions

In conclusion, 8 days of either L-Cit or L-Arg supplementation did not enhance middle (200 m) and short distance (100 m) swimming performance in trained/developmental swimmers and triathletes. These findings do not support the use of L-Cit or L-Arg supplementation as ergogenic aids for swimming performance.

## Figures and Tables

**Table 1:** Group mean (SD) of NOx and BLa responses after 8-day L-citrulline or L-arginine or placebo supplementation. PLA, placebo; ARG, L-arginine; CIT, L-citrulline; Con, control trial; Exp, experimental trial; BLa, blood lactate.

	PLA (*n* = 5)		ARG (*n* = 5)		CIT (*n* = 5)	
	*Con*	*Exp*	*Con*	*Exp*	*Con*	*Exp*
**NOx** (nmol/L^−1^)	66.2 ± 22.1	59.4 ± 13.6	44.1 ± 19.3	66.4 ± 6.7	42.5 ± 1.2	58.4 ± 7.3
**200 m TT** (s)	175.20 ± 24.81	176.16 ± 25.87	152.33 ± 19.24	149.52 ± 15.34	156.47 ± 10.79	155.30 ± 8.76
**100 m TT** (s)	82.06 ± 12.55	81.01 ± 11.45	67.97 ± 06.91	66.97 ± 05.89	70.35 ± 05.21	69.19 ± 04.40
**BLa Pre-200 m** (mmol/L^−1^)	3.2 ± 1.4	3.5 ± 1.5	4.0 ± 2.3	3.9 ± 2.0	3.2 ± 0.9	3.1 ± 1.1
**BLa post-200 m** (mmol/L^−1^)	10.3 ± 2.0 *	11.2 ± 1.2 *	12.5 ± 2.0 *	13.4 ± 2.2 *	14.1 ± 2.7 *	11.3 ± 0.9 *
**BLa pre-100 m** (mmol/L^−1^)	4.0 ± 1.0	4.7 ± 1.3	7.2 ± 2.6	8.0 ± 2.2	6.7 ± 2.7	5.4 ± 1.1
**BLa post-100 m** (mmol/L^−1^)	10.3 ± 1.6 *	10.2 ± 0.6 *	14.0 ± 2.2 *	14.7 ± 1.9 *	13.8 ± 2.8 *	12.6 ± 1.3 *

* Statistically significant difference between pre and post values (*p* < 0.001).

## Data Availability

The data presented in this study are available on request from the corresponding author. The data are not publicly available due to restrictions privacy.
